# Comparative Evaluation of Sorption and Solubility of Core Buildup Materials in Different pH Media: An In-Vitro Study

**DOI:** 10.7759/cureus.59432

**Published:** 2024-05-01

**Authors:** Waseem Khan, Kapilesh B Singh, Damini Patil, Anik Banerjee, Chetan D Ahire, Vikas Singh

**Affiliations:** 1 Conservative Dentistry and Endodontics, Yogita Dental College and Hospital, Khed, IND; 2 Conservative Dentistry and Endodontics, SMBT Dental College & Hospital, Sangamner, IND; 3 Conservative Dentistry and Endodontics, Kothiwal Dental College & Research Centre, Moradabad, IND; 4 Conservative Dentistry and Endodontics, Karmaveer Bhausaheb Hiray Dental College & Hospital, Nashik, IND; 5 Public Health Dentistry, Teerthanker Mahaveer Dental College & Research Centre, Moradabad, IND

**Keywords:** core build-up materials, materials, dentistry, sorption, solubility

## Abstract

Aim: To evaluate and compare the sorption and solubility of two different core buildup materials in different pH media for periods of one day, one week, and one month.

Materials and method: Sixty samples were prepared and divided into Group A (30 resin-based samples) and Group B (30 glass ionomer cement (GIC)-based samples). The sorption and solubility of the different materials were calculated by weighing the samples before and after desiccation and media immersion for periods of one day, one week, and one month. Groups were compared using the Mann-Whitney U test, and for different media, the intragroup significance of the mean difference was performed using the Friedmann test and Wilcoxon signed rank test at a significance level of p<0.05.

Results: After immersion for different time periods, the resin-based core buildup material (Core X flow) showed less sorption and solubility as compared to the glass ionomer-based core buildup material (Secure Core Z) for all time periods, with a significant difference seen for a time period of one week and one month and being nonsignificant for a time period of one day.

Conclusion: Core X flow had lower sorption and solubility values when compared to Secure Core Z, as per the International Organization for Standardization (ISO) 4049 standards, except for a one-month time period in alkaline media.

## Introduction

An ideal core buildup material should have physical characteristics similar to those of the tooth structure [1]. The clinical success and longevity of core buildup materials depend on different properties, such as structural integrity and dimensional stability, which are in turn functions of sorption and solubility. Sorption and solubility can affect mechanical strength, color stability, and abrasion resistance [2,3]. Restoration failure is caused by the loss of surface characteristics, aesthetics, and marginal integrity, which is a result of both sorption and solubility [4]. Dimensional changes, swelling, and hygroscopic expansion are caused by moisture in the oral environment [5].

Prior to the tooth preparation procedure in the following visit, which could be delayed for several weeks or months, resin-based core buildup materials are frequently left exposed in the oral cavity. These materials are typically used to fill extensively damaged tooth structures in conjunction with prefabricated fiber posts. In this type of clinical scenario, resin-based core buildup materials may inevitably be exposed to a moist environment for an extended period of time, resulting in increased chances of sorption and solubility [6-8].

It is widely known that when these materials are utilized for either core buildup or adhesive bonding, the hygroscopic expansion of resin-modified glass ionomers and compomers can result in the failure of all ceramic crowns [3]. The susceptibility of resin-based materials to sorption may lead to an expedited deterioration process that promotes the degeneration of polymer resin and has significant implications for the long-term durability of restorations. The sorption and solubility behaviors are significantly influenced by factors such as the type of resin composite material, chemical composition, storage time, type and pH of the storage solution, and degree of polymerization [9].

When the resin samples are immersed in water, two opposing phenomena occur. On the one hand, water leaches out unreacted monomers and other species, contributing to shrinkage, loss in weight, and reduction in mechanical properties. However, sorption causes the material to swell, followed by an increase in weight. An in vitro study showed that restorations cemented to extracted teeth that were exposed to deionized water eventually fractured [10-14].

The majority of earlier studies examined the sorption and solubility of polymeric substances such as acrylic denture base resins, soft lining resins, and composite resins in various immersion media such as water, artificial saliva, and ethanol. However, few studies have evaluated the effect of acids produced by human dental plaque, such as lactic acid. Studies on the action of this acid on newly developed core buildup materials may increase our knowledge regarding their durability in the oral environment [14,15].

To date, there are very few reports in the literature on the sorption and solubility of core buildup materials, especially in different pH media, as there is a constant change in pH in the oral cavity during different time periods in a day. Therefore, the purpose of this in vitro study was to evaluate and compare the sorption and solubility of different core build-up materials in different pH media.

The null hypothesis is that there is no difference between the sorption and solubility of one resin-based core buildup material and one glass ionomer cement (GIC)-based zirconia-reinforced core buildup material for different time periods.

## Materials and methods

The materials used in the study included core buildup materials for sample preparation, such as acetic acid and sodium acetate for the acidic buffer solution and sodium carbonate and sodium bicarbonate for the alkaline buffer solution. There is sorption and solubility of core buildup materials in different pH media for different periods with higher changes seen in GIC-based core buildup materials. Sodium hydroxide and hydrochloric acid were used to maintain the pH of the buffer solutions, while distilled water was used for the preparation of neutral media. Several chemical agents such as acetic acid, sodium bicarbonate, etc., along with proper equipment, such as measuring cylinders, beakers pipettes, etc., were used. The pH values were ensured with a digital pH meter with proper calibration. The pH values were 5, 7, and 9 for acidic, neutral, and alkaline media, respectively. They were placed in an incubator with temperature adjusted to around 37 degrees centigrade (Table 1).

**Table 1:** Materials used in the study

S. No.	Product	Batch No.	Company
1.	Core-X flow	2103000670	Dentsply
2.	Secure core Z	072013-81	Wizdent
3.	Acetic acid and sodium acetate buffer tablets	3591590819	Thermofisher Scientific India
4.	Sodium carbonate and sodium bicarbonate buffer tablets	2809	Metal Technologies
5.	Sodium hydroxide flakes	24350	Labogens Corporation
6.	Hydrochloric acid solution	44321	Labogens Corporation
7.	Distilled water	20525	RPI (Research Products International)

**Figure 1:**
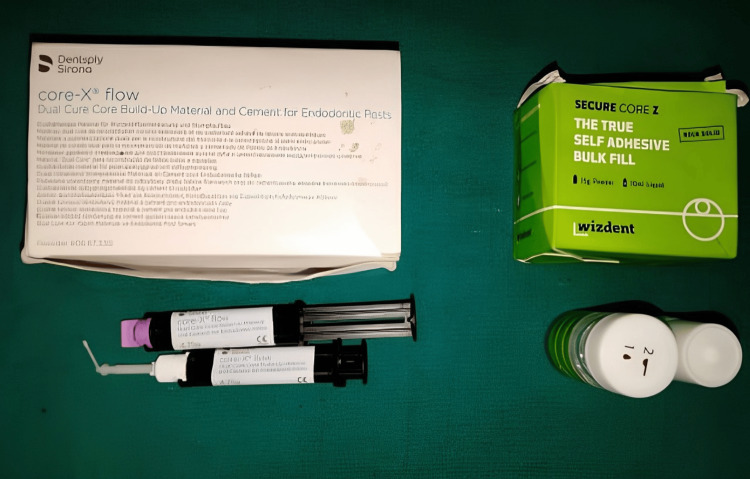
Materials used for the study: Core X flow and Secure Core Z

The armamentarium consisted of various apparatus and equipment such as desiccators (for providing a dry environment), incubators (for maintaining temperature), and analytical scales (for measuring weight) essential for the fabrication and testing of the samples (Figure 1).

**Figure 2:**
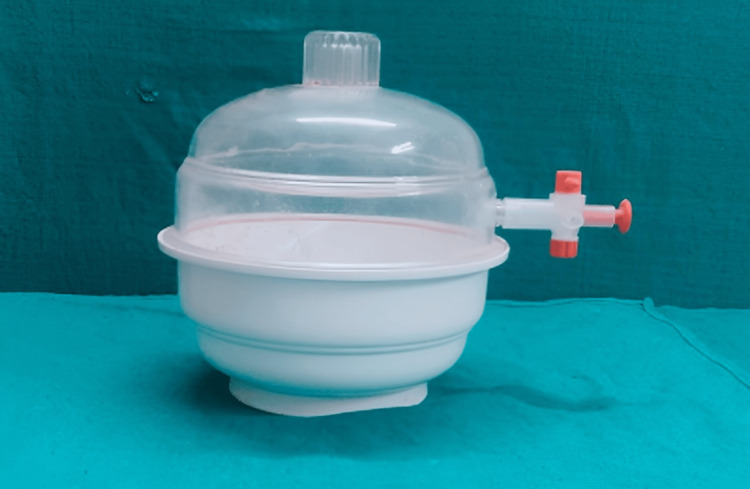
Incubator used in the study

For sample fabrication (light cured from top and bottom as samples were placed between glass plates, and sufficient time was given for letting them cure chemically), a stainless steel split ring mold with specified dimensions (15 ± 0.1 mm in diameter and 1.0 ± 0.1 mm deep) was utilized, along with glass plates to cover each side of the mold and ensure a smooth surface finish. A polyester sheet (50 ± 30 μm thick) was employed for the easy separation of the samples, while Vaseline served as a separating medium between the samples and the metal mold. Desiccators were utilized to provide a dry environment, and an incubator was employed to maintain a consistent temperature conducive to sample testing (Figure 2).

**Figure 3:**
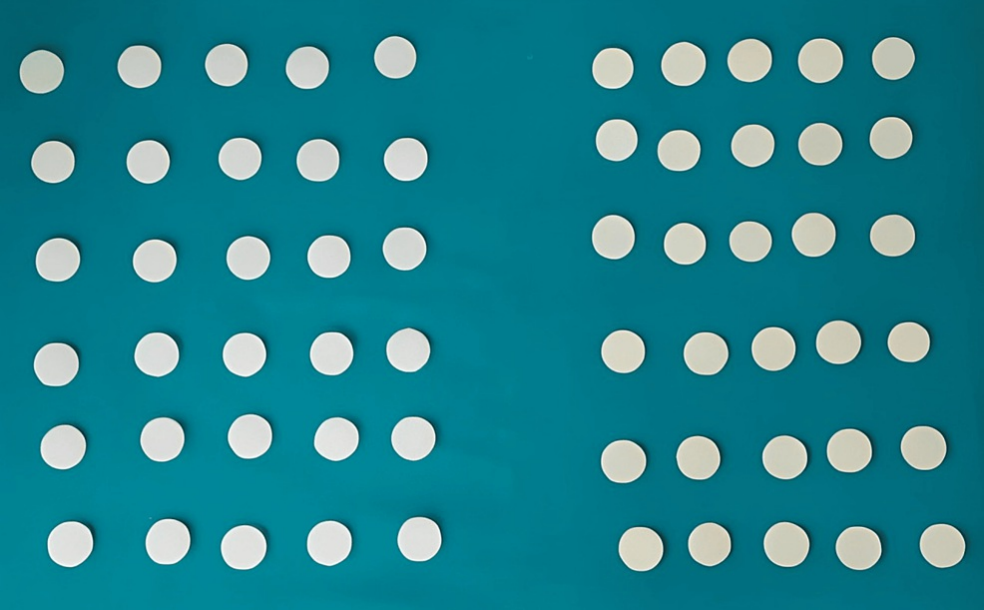
Materials were divided into various groups in the study

For the testing of the samples, an analytical scale was used to measure the weight accurately, ensuring precision in the experimental procedures. This study was conducted to compare and evaluate the solubility and sorption of core buildup materials after immersion in three media with different pH values. In the present study, 60 samples were fabricated, which were divided into Group A (30 resin-based samples) and Group B (30 GIC-based samples). The sample size was calculated using G power software (The G*Power Team, Germany). Based on the calculated effect size of 0.396, 5% level of precision, 95% confidence level, and 80% power of the study, the sample was rounded off to 60 with 30 in each group (Figure 3).

For solubility and sorption (Wsolubility = W1-W3/V and Wsorption = W2-W3/V), the specimens were transferred to one of two desiccators maintained at 37°C. After 22 hours, the specimens were removed and stored in a second desiccator, maintained at 23°C for two hours, and then weighed to an accuracy of 0.1 mg. This cycle was repeated until a constant mass W1 was obtained. The area was calculated in square millimeters from the mean diameter, and then using the mean thickness, the volume (V) was calculated in cubic millimeters. The specimens were immersed in media (acidic, alkaline, and neutral) in beakers at 37°C for one day and seven and 30 days. The incubator was maintained at a temperature of 37°C. The volume of water for the immersion of the specimens should not be less than 10 mL.

After different immersion periods, the specimens were removed, washed with water, blotted away with surface water until they were free from visible moisture, waved in air for 15 seconds, and weighed one minute after removal from water. The mass was recorded as W2. The specimens were reconditioned to a constant mass in a desiccator. Constant mass was recorded as W3. The values were calculated for solubility, Wsl, and sorption (Wsorp) in micrograms per cubic millimeter. Data were analyzed using Statistical Product and Service Solutions (SPSS, version 21.0; IBM SPSS Statistics for Windows, Armonk, NY). Groups were compared using the Mann-Whitney U test. The level of statistical significance was set at p < 0.05.

## Results

Table 2 presents a comparative analysis of the sorption levels between two groups, Group A and Group B, at different time intervals (T1, T2, and T3) and under varying pH conditions (acidic, neutral, and alkaline).

**Table 2:** Comparison of sorption at all intervals of time between Group A and Group B SD: standard deviation The level of statistical significance was set at a p-value less than 0.05.

Sorption		Group A		Group B		Mean difference	P value
		Mean	SD	Mean	SD		
T1 (1 day)	Acidic	1.66	0.19	4.74	1.01	-3.08	0.458
	Neutral	1.63	0.24	4.28	0.71	-2.64	0.45
	Alkaline	3.64	0.47	6.18	0.44	-2.55	0.11
T2 (1 week)	Acidic	15.78	0.82	34.79	0.93	-19.01	0.001
	Neutral	17.78	0.68	33.08	1.38	-15.30	0.001
	Alkaline	32.40	0.55	51.69	1.40	-19.29	0.001
T3 (1 month)	Acidic	37.41	0.43	64.54	1.02	-27.14	0.001
	Neutral	25.66	0.72	56.23	1.41	-30.56	0.001
	Alkaline	57.42	0.95	103.29	1.34	-45.87	0.001

At T1, there were no significant differences in sorption between the two groups across all pH levels. However, significant differences were observed at time intervals T2 and T3. Specifically, Group B consistently exhibited higher sorption levels than Group A pH environments, with p-values indicating statistical significance (p < 0.001). For instance, in the acidic environment at T2, Group B showed a mean sorption of 34.79 (SD = 0.93), significantly higher than Group A's mean sorption of 15.78 (SD = 0.82). This pattern persisted across all time intervals and pH levels, highlighting the consistent trend of higher sorption in Group B. These findings suggest a notable impact of the group variable on sorption outcomes, with Group B consistently demonstrating elevated sorption levels compared with Group A.

Table 3 presents a detailed comparison of solubility between Groups A and B at various time intervals.

**Table 3:** Comparison of solubility at all intervals of time between Group A and Group B SD: standard deviation

Solubility		Group A		Group B		Mean difference	p value
		Mean	SD	Mean	SD		
T1 (1 day)	Acidic	1.65	0.14	2.97	0.61	-1.32	0.541
	Neutral	1.22	0.09	3.09	0.40	-1.86	0.301
	Alkaline	2.22	0.20	3.98	0.52	-1.75	0.542
T2 (1 week)	Acidic	3.80	0.18	8.13	0.48	-4.33	0.01
	Neutral	2.83	0.22	7.67	1.03	-4.83	0.01
	Alkaline	6.41	0.42	13.81	0.79	-7.39	0.01
T3 (1 month)	Acidic	6.80	0.17	14.34	0.71	-7.53	0.01
	Neutral	6.63	0.32	8.42	0.55	-1.78	0.01
	Alkaline	13.78	0.55	25.52	0.89	-11.74	0.01

Mean solubility values and their SD are provided for each group and condition. Moreover, the table offers an insight into the mean difference and corresponding p-values for each comparison. At the initial time interval (T1), no statistically significant differences in solubility were observed between Groups A and B under acidic, neutral, and alkaline conditions (p > 0.05). The mean solubility values for Group A ranged from 1.22 to 2.22, with an SD between 0.09 and 0.20, while for Group B, they varied from 2.97 to 3.98, with an SD between 0.40 and 0.61. However, as the observation progressed to T2 and T3, notable differences in solubility emerged between the two groups across all acidic, neutral, and alkaline conditions. At T2, Group B exhibited significantly higher solubility than Group A, with mean differences ranging from -4.33 to -7.39 (p < 0.01). Similarly, at T3, Group B continued to demonstrate significantly higher solubility values, with mean differences ranging from -7.53 to -11.74 (p < 0.01). These findings highlight a clear trend wherein the Group B materials displayed consistently greater solubility than the Group A materials as the observation period progressed. Such distinctions underscore the potential disparities in material properties and compositions between the two groups, suggesting implications for their practical applications and durability over time.

## Discussion

In this study, the sorption and solubility characteristics of core buildup materials, including one resin-based and one GIC-based material, were assessed over a period of one day, one week, and one month in alkaline, neutral, and acidic media. Differences based on the type of storage medium were significant for GIC-based materials and comparatively less for resin-based core buildup materials. Sorption and solubility tests, as used in this study, involved the placement of disc specimens in the respective media for different time periods. Adsorption and absorption are both included in sorption properties [14,16].

The acidic-alkaline pH of the oral cavity varies in relation to the foods consumed, as well as the salivary changes in each individual [17]. GIC that are conventionally and light-cured are known to absorb water and may disintegrate through surface wash-off, diffusion through pores and fissures in the cement, or diffusion from the bulk; however, the new light-curing GIC have improved properties in the initial phase of setting, including reduced early solubility [2,4,18]. Some glass ionomer components have also been combined with zirconia, known as zirconia reinforcement, and some with resin composite substances. These polyacid-modified resin composites are intended to combine the advantages of both types of filling materials but lack the typical glass ionomer acid/base reaction during the initial setting process. Zirconia also exhibits adequate chemical and dimensional stability, which prevents it from dissolving during an increasing soaking period [14,18,19].

A previous study [5] investigated the variation in water sorption and solubility of five core buildup materials, including four resin-based materials (Grandio Core, Core X flow, Bright Flow Core, and Speedee) and one resin-modified glass ionomer (Fuji II LC). The results showed that Grandio Core exhibited the lowest water sorption and solubility among the tested materials. According to the International Organization for Standardization (ISO) standards, all the tested materials showed acceptable water sorption and solubility, apart from the water sorption behavior of Fuji II LC [5]. Several studies have been performed to test the behavior of dental materials that use water, acids, and other solvents to simulate the contaminating environment of the mouth [9,20]. The chemical structures of the solutions used for in vitro tests are important for simulating the complexity of the oral environment [21].

In the present study, the two materials tested exhibited high sorption and solubility values in an alkaline medium. In the case of Secure Core Z, the sorption and solubility values in all three media exceeded the maximum acceptable values for GIC-based restorative materials after one week and one month, except for sorption for one week in neutral and acidic media and solubility in neutral medium. For Core X flow, the values were within the limits of acceptable values (7.5 g/mm^3^ for solubility and 40 g/mm^3^ for sorption), except for sorption and solubility for a one-month period in an alkaline medium. Water sorption is a diffusion-controlled process that occurs in organic resin matrices and depends mainly on the resin composition [22]. The observed differences in water uptake can be attributed to the nature of the filler particles and coupling agents. Therefore, sorption should be significantly reduced at the interface in systems where the matrix and filler particles are effectively coupled. The hydrophilicity of the monomers used in resin-based materials plays a major role in their sorption. The resin-based materials tested showed lower water sorption values than the zirconia-reinforced glass ionomer. This may be attributed to the fact that their matrix is more hydrophobic than that used in GIC-based materials. The water uptake in resin composites occurs in the matrix, whereas in the glass ionomer, the impact is on the hydrogel structure, which leads to increased water uptake [5,23]. GIC have the major drawback of moisture sensitivity until the completion of the setting reaction. This could be related to the present study from high values of water sorption and solubility of the glass ionomer-based core buildup material in all three immersion media. This is due to the fact that water is absorbed as a result of cement-forming cations being eluted. Water penetration has, however, diminished as the cement structure continues to grow. The mixing process could result in air spaces, which could hasten water sorption and solubility. Glass-ionomer surface hardness may decrease as a result of matrix dissolution brought on by the loss of the siliceous hydrogel. These glass ionomer material specimens were not protected after being set up with a hydrophobic layer. This may also be a reason for their high values. Hydrophilic constituents such as urethane dimethacrylate (UDMA) (present in Core X flow) or resin molecules that contain hydrophilic moieties clearly increase water sorption values [24].

The results of sorption and solubility studies during different time intervals and pH conditions are of great clinical importance, especially in dental materials science when comparing Group A and Group B. In both sorption and solubility tests, Group B consistently reported higher values over time compared to Group A, showing that the materials used in Group B may have different or improved features influencing its behavior during installation in a biological environment. This is very critical in dental practices that demand materials to stand up in diverse and dynamic oral conditions. This means, therefore, that dental restorations may deteriorate and wear with time due to the degradation of materials with higher sorption and solubility rates found in Group B, hence affecting longevity and effectiveness. Therefore, knowledge of such properties could help in the selection of materials for various dental applications with a view to improving the longevity and quality of restorative treatments. Further implications may be derived in formulating and improving the dental materials from these tests. For example, materials with low sorption and solubility under all pH conditions, such as those in group A, would likely be more accepted for those environments in which stability and resistance of oral fluids are required. This, in fact, could lead to the development of a material that is durable and compatible with the physiological conditions prevailing in the mouth. This information could be used by dental professionals and researchers to be extrapolated in an effort to predict behavior under real-life conditions and help make decisions about the material selection for a dental restoration a more informed one. Such research further emphasizes the need for continued material testing to guarantee safety and efficacy, aligning the development of dental material to the growing requirements of material usage in clinical dentistry and patient care.

It can be concluded from the results of this study that the sorption and solubility were significantly higher for the glass inomer-based core buildup material than for the resin-based core buildup material in all three media, with a significant difference observed during the time periods of one week and one month and a nonsignificant difference for the time period of one day. A comparable performance between bulk-fill composites (BFCs) and progressively inserted conventional resin composites (CRC) has been observed in previous studies [25]. The present study found that the Core X flow had lower sorption and solubility values when compared to Secure Core Z as per ISO 4049 standards, except for a one-month time period.

To address the methodological limitations of the study, a more comprehensive analysis of potential confounding factors and sources of bias is essential. The study's reliance on a small, possibly non-representative sample limits its ability to capture the full variability of the materials, thus affecting the generalizability of the findings. Additionally, the absence of longer-term data restricts understanding of the materials' performance over extended periods and under variable conditions. To mitigate these issues, future studies should consider increasing sample sizes and incorporating diverse material types to enhance representation. Furthermore, extending the duration of observations and including varying environmental conditions, such as different pH levels, could provide more robust data on the materials' behavior and stability. This approach would help in identifying and adjusting for confounding variables that could skew the results, thereby enhancing the study's credibility and relevance to clinical practice. Regarding the transparency and reproducibility of research findings, to improve the integrity of future research, adopting practices such as pre-registering the study protocol and conducting comprehensive literature searches before experimental initiation is crucial.

## Conclusions

The study concluded that the Core X Flow had lower sorption and solubility values when compared to Secure Core Z as per ISO 4049 standards, except for a one-month time period in alkaline media, where it was beyond the acceptable value of ISO 4049. On the other hand, Secure Core Z crossed the ISO 4049 standards for one week and one month for all media.
